# Do Reading and Arithmetic Fluency Share the Same Cognitive Base?

**DOI:** 10.3389/fpsyg.2021.709448

**Published:** 2021-07-28

**Authors:** George K. Georgiou, Tomohiro Inoue, Rauno Parrila

**Affiliations:** ^1^Department of Educational Psychology, University of Alberta, Edmonton, AB, Canada; ^2^Department of Psychology, The Chinese University of Hong Kong, Hong Kong, China; ^3^School of Education, Macquarie University, Sydney, NSW, Australia

**Keywords:** reading fluency, arithmetic fluency, rapid automatized naming, phonological awareness, speed of processing, working memory, number sense

## Abstract

We examined the role of different cognitive-linguistic skills in reading and arithmetic fluency, and whether the effects of these skills are mediated by reading and arithmetic accuracy. One hundred twenty-six English-speaking Grade 1 children (67 females, 59 males; *M*_age_ = 6.41 years) were followed from the beginning of Grade 1 (Time 1) to the end of Grade 1 (Time 2). At Time 1, they were assessed on measures of non-verbal IQ, speed of processing, working memory, phonological awareness, rapid automatized naming (RAN), and number sense. At Time 2, they were assessed on measures of reading and arithmetic accuracy as well as on measures of reading and arithmetic fluency. Results of path analysis showed first that when reading and arithmetic fluency were included in the model as separate outcomes, RAN was predictive of both and that speed of processing and working memory were predictive of only arithmetic fluency. Second, RAN, speed of processing, and working memory had both direct and indirect effects (*via* reading and arithmetic accuracy) on the covariation of reading and arithmetic fluency. Irrespective of how reading and arithmetic fluency were treated in the analyses, the effects of non-verbal IQ, phonological awareness, and number sense were all indirect. Taken together, these findings suggest that reading and arithmetic fluency draw on a broader network of cognitive-linguistic skills, whose effects can sometimes be indirect through reading and arithmetic accuracy.

## Introduction

For decades, research on the predictors of reading and mathematics skills has focused on each academic skill separately. Cognitive skills such as phonological awareness (the ability to identify and manipulate the speech sounds) and rapid automatized naming (RAN; the ability to name as fast as possible highly familiar stimuli) have been described as fundamental for learning to read (e.g., Hulme and Snowling, 2015). Likewise, number sense (an intuitive understanding of numbers, their magnitude, and relations) and counting have been viewed as critical for the development of mathematics skills (e.g., Geary, 2011). However, recent cross-domain research has revealed that there is considerable overlap in the predictors of reading and mathematics skills (e.g., Koponen et al., 2007, 2016, 2020; Korpipää et al., 2017; Purpura et al., 2017; Cirino et al., 2018). For example, in one of the pioneering studies, Koponen et al. (2007) showed that counting (assessed in kindergarten) and RAN (assessed in Grade 4) were significant predictors of both reading and arithmetic fluency in Grade 4.

Despite the recent proliferation of cross-domain studies examining the role of different cognitive predictors of reading and mathematics skills, several issues remain unclear. First, only a handful of studies have examined the predictors of the shared variance (i.e., covariation) between reading and mathematics skills, they have all been conducted in Finnish (a transparent alphabetic orthography), and have focused on fluency (e.g., Koponen et al., 2013, 2020; Korpipää et al., 2017). Given the possible impact of orthographic transparency on reading development and its predictors (e.g., Georgiou et al., 2008; Moll et al., 2014), their findings need to be replicated in orthographies that are less transparent than Finnish. Second, to our knowledge, none of the previous studies that examined the role of different cognitive skills in the covariation of reading and arithmetic fluency have examined if the effects of these predictors are mediated by reading and mathematics accuracy. Finally, with one exception (see Koponen et al., 2020), all previous studies that examined the predictors of the covariation of reading and mathematics skills have focused on counting as a math-related skill (e.g., Koponen et al., 2007, 2013, 2016; Korpipää et al., 2017). Thus, we do not know if other basic number skills (e.g., number sense) are also important. To address these shortcomings, we aimed to examine if reading-related skills (phonological awareness and RAN), math-related skills (number sense), and general cognitive abilities (non-verbal IQ, speed of processing, and working memory) account for the covariance between reading and arithmetic fluency in English, an opaque alphabetic orthography, and if the effects of these skills are mediated by the effects of reading and arithmetic accuracy.

### The Predictors of the Covariation Between Reading and Arithmetic

Several studies have shown that reading and mathematics are highly correlated (e.g., Koponen et al., 2007; Landerl and Moll, 2010; Codding et al., 2015; Balhinez and Shaul, 2019; Erbeli et al., 2020), and that comorbid disabilities occur far more often than isolated reading, and mathematics disabilities (e.g., Dirks et al., 2008; Willcutt et al., 2013; Koponen et al., 2018). Researchers have also argued that the observed covariation of reading and mathematics skills may be partly due to the fact that the development of both academic skills relies on similar cognitive processes (e.g., Koponen et al., 2007, 2020; Zoccolotti et al., 2020). Thus, examining the predictors of the covariation can reveal important information about the cognitive base of reading and mathematics acquisition.

Two slightly different approaches have been used to examine the unique and shared predictors of reading and mathematics skills. First, some researchers have created a latent factor to represent the shared variance between reading and mathematics skills and then regressed that factor on different predictors (Koponen et al., 2007, 2013, 2016, 2020; Korpipää et al., 2017). This makes sense if we are looking at what cognitive processes underlie what is common between reading and mathematics, but, at the same time, it does not allow us to say what processes are unique predictors of each academic skill. For example, it is possible that a cognitive process might be a significant predictor of reading, but not of what is shared between reading and mathematics. The second approach might be considered a mirror image of the first. Researchers have included both reading and mathematics tasks as dependent variables in the same model (allowing them to co-vary), and then used several predictors to examine which ones predict both outcomes and which ones predict only reading or mathematics (e.g., Slot et al., 2016; Hornung et al., 2017; Peterson et al., 2017; Yang et al., 2021). Even though this approach can show us what cognitive processes predict each outcome measure, it does not tell us if they predict the covariation between the two outcomes. For this reason, we employed both approaches in our study.

Obviously, an important question in this line of research is what cognitive processing skills are included as predictors. Previous studies have considered three kinds of skills: linguistic skills (e.g., Cirino et al., 2018; Zhang and Lin, 2018; de Megalhães et al., 2021), basic number skills (e.g., Koponen et al., 2016, 2020; Cirino et al., 2018), and general cognitive abilities (e.g., Cattell, 1987; Gathercole et al., 2004; Alloway and Alloway, 2010; Georgiou et al., 2015). In regard to the linguistic skills, researchers have focused mostly on the role of phonological awareness and RAN, both of which are considered components of phonological processing (e.g., Wagner and Torgesen, 1987). Phonological awareness is important for learning to read because it is involved in matching the letters (i.e., graphemes) in words to their corresponding sounds (i.e., phonemes) and supports the blending of the sounds in word recognition. Likewise, it is important in mathematics because some mathematics tasks (e.g., counting) involve processing of verbal codes (see triple-code model of numerical cognition; Dehaene, 1992; Dehaene et al., 2003; see also De Smedt et al., 2010). More specifically, when asked to solve a mathematics problem, children may convert the terms, operators, and quantities into sound-based codes and unimpaired access to these codes can support the execution of the problems. However, evidence from previous studies is mixed. Whereas, some cross-domain studies have reported significant effects of phonological awareness in both reading and mathematics (e.g., Slot et al., 2016; Cirino et al., 2018; Zhang and Lin, 2018; de Megalhães et al., 2021), others have reported significant effects only on reading (e.g., Durand et al., 2005; Peterson et al., 2017) or no significant effects on either academic skill (e.g., Yang et al., 2021). Studies on the predictors of the shared variance between reading and mathematics skills have also reported mixed findings. Whereas, Korpipää et al. (2017) found that phonological awareness (measured with an initial sound identification task at the Fall of Kindergarten) was not a significant predictor of the time-invariant covariation between reading and arithmetic fluency,^1^ Koponen et al. (2020) found that phonological awareness (measured with a syllable and phoneme deletion task at the Spring of Grade 1) was a significant predictor of the covariation between reading and arithmetic fluency at the Fall of Grade 2.

Beyond phonological awareness, researchers have also examined the role of RAN in both reading and mathematics skills (particularly arithmetic fact fluency; e.g., Koponen et al., 2007, 2013, 2016, 2020; Georgiou et al., 2013; Hornung et al., 2017; Balhinez and Shaul, 2019). For example, in a longitudinal study with Finnish children followed from kindergarten to Grade 3, Koponen et al. (2016) found that RAN was a significant predictor of both reading and arithmetic fluency, even after controlling for the effects of phonological awareness, verbal short-term memory, vocabulary, counting, and mother's education.

Examining the relation between RAN and reading/mathematics skills in the same study is interesting in light of theoretical accounts that have been put forward to explain their relation. For example, (e.g., Wagner and Torgesen, 1987; Torgesen et al., 1994, 1997) have argued that RAN reflects the speed of access to, and retrieval of, phonological representations from long-term memory. If phonological representations are of low quality, this will interfere with the retrieval, manipulation, and retention of phonological codes, which, in turn, will impede reading development. However, researchers have also argued that if phonological representations for number words and number facts in long-term memory are weak, this will affect how quickly they can be retrieved from long-term memory, which, in turn, will impact mathematics development (e.g., Simmons and Singleton, 2008; De Smedt et al., 2010). To the extent the conceptualization of RAN as an index of children's ability to access and retrieve phonological representations from long-term memory is correct, RAN should predict the covariation of reading and mathematics skills (at least of tasks such as word reading fluency and addition fluency that rely on quick access to phonological representations in long-term memory). Koponen et al. (2016, 2020) findings are in line with this prediction.

Examining the role of RAN in the covariation of reading and mathematics skills is also interesting because some math researchers have used RAN tasks as measures of speed of processing (e.g., Berg, 2008; Chan and Ho, 2010; Vanbinst et al., 2015). Kail and colleagues (Kail and Hall, 1994; Kail et al., 1999) have also argued that speed of processing is *per se* important in tasks such as reading and mathematics that require timely integration of information within and between cognitive sub-processes. If RAN is a measure of speed of processing, then it should predict the shared variance between reading and arithmetic fluency tasks because both outcomes are speeded. If this is the case, then RAN's effects on the covariation should also disappear after controlling for other measures of speed of processing. Existing research has shown that controlling for speed of processing accounts for only a small part of the RAN-reading relation (e.g., Bowey et al., 2005; Georgiou et al., 2016); if RAN specifically captures access to the phonological representations for number words and facts, the same should be true for arithmetic fluency. This, however, may not be the case: in a study with Greek-speaking children, Georgiou et al. (2013) showed that speed of processing was enough to eliminate RAN's effects on arithmetic fluency, but not on reading fluency, suggesting that different mechanisms account for RAN-reading and RAN-arithmetic fluency connections.

Beyond the linguistic skills, basic number skills (e.g., counting, number sense) may be associated with the covariation of reading and mathematics skills. Most previous studies have focused on counting (Koponen et al., 2007, 2016, 2020; Korpipää et al., 2017). Koponen et al. (2007), for example, showed that counting (measured in kindergarten) was a significant predictor of the covariation of single-digit calculation and text reading in Grade 4 over and above the effects of letter knowledge and RAN. Koponen et al. (2020) further showed that a latent factor consisting of counting and RAN in Grade 1 (called “serial retrieval fluency”) was a significant predictor of the covariation of reading and arithmetic fluency in Grade 2 over and above the effects of letter knowledge, phonological awareness, number comparison, and number writing. Interestingly, number comparison and number writing also predicted the covariation. To our knowledge, no studies have examined the role of number sense in the covariation of reading and mathematics skills. However, in a cross-domain study with 130 Grade 1–5 Dutch children, Slot et al. (2016) found that number sense was predictive of only mathematics skills. Thus, in this study we aimed to replicate this finding.

Finally, general cognitive abilities, such as non-verbal IQ, speed of processing, and working memory may predict the covariation of reading and mathematics skills. In regard to non-verbal IQ, several studies have shown that it is associated with both academic skills (e.g., Deary et al., 2007; Roth et al., 2015; Peng et al., 2019). For example, in their meta-analysis, Peng et al. (2019) estimated the average correlation between non-verbal IQ (fluid intelligence) with reading and mathematics to be 0.38 and 0.41, respectively. Korpipää et al. (2017) also showed that non-verbal IQ was a significant predictor of the time-invariant covariation of reading and arithmetic fluency; a finding that needs replication. In regard to working memory, researchers have argued that it is particularly important for reading comprehension (Kendeou et al., 2014) because children must retain information in their short-term memory while processing other parts of text. However, it may also contribute to word recognition because young children may hold the sound of individual letters in their memory while visually processing the upcoming letters within a word before blending of the individual sounds takes place. Likewise, it is needed when solving different mathematics problems [e.g., (3 + 6) ^*^ 6 = ?] because individuals need to first hold part of the solution in their memory (e.g., the result of 3 + 6) before executing another operation (e.g., multiplying by 6). However, evidence on the role of working memory in reading and mathematics skills is mixed (e.g., Alloway and Alloway, 2010; Peterson et al., 2017; Balhinez and Shaul, 2019; de Megalhães et al., 2021; Yang et al., 2021). For example, Balhinez and Shaul (2019) showed that working memory was a significant predictor of both reading and arithmetic fluency in Grades 1 and 2. In turn, working with a sample of Grade 5 and 5 children, de Megalhães et al. (2021) showed that working memory was a significant predictor of arithmetic accuracy and fluency, but not of reading accuracy and fluency. Finally, Yang et al. (2021) showed that working memory was not a significant predictor of either reading or mathematics skills in Grade 1. Clearly, more research is needed on the role of working memory in the covariation of reading and mathematics skills.

To summarize, even though a few studies have examined the role of different cognitive processes in reading and mathematics skills in the same study (e.g., Hornung et al., 2017; Peterson et al., 2017; Cirino et al., 2018; Balhinez and Shaul, 2019; Yang et al., 2021), very few have examined the predictors of the covariation of reading and mathematics skills (Koponen et al., 2007, 2016, 2020; Korpipää et al., 2017). Given that both the dependent variables and the predictors are measured with complex, multifaceted tasks, failing to take the covariance between reading and mathematics skills into account does not allow us to draw firm conclusions on the shared cognitive base of these academic skills.

### The Present Study

The present study aimed to answer the following two research questions:

1. To what extent do linguistic skills (phonological awareness and RAN), number skills (number sense), and general cognitive abilities (non-verbal IQ, speed of processing, and working memory) predict reading and arithmetic fluency, and their covariation? Based on the findings of previous studies (e.g., Koponen et al., 2007, 2020; Korpipää et al., 2017), we expected that RAN would be a significant predictor of both academic skills as well as of their covariation. Because previous studies have provided mixed findings for the rest of the predictors, we did not formulate any specific hypotheses for them.
2. To what extent the effects of the linguistic skills, number skills, and general cognitive abilities on the covariation of reading and arithmetic fluency will be mediated by the effects of reading and arithmetic accuracy? We did not formulate any specific hypotheses here because no previous studies have examined the role of reading/arithmetic accuracy in these relations.

The findings of this study are expected to contribute to the literature in two important ways. First, as mentioned above, findings on the predictors of the covariation of reading and arithmetic fluency need to be replicated in a language with a less transparent orthography. This not only allows us to validate the previous findings, but also to examine the possible mediating role of reading and mathematics accuracy. Because reading accuracy reaches ceiling by the end of Grade 1 in Finland (Seymour et al., 2003), this may have prevented Koponen et al. (2007, 2016, 2020) and Korpipää et al. (2017) from testing the mediating role of reading accuracy. Given that RAN and number sense are related to reading and arithmetic accuracy (e.g., Leppänen et al., 2004; Slot et al., 2016; Zhang and Lin, 2018) and reading and arithmetic accuracy are significant predictors of reading and arithmetic fluency (e.g., Nunes et al., 2012; Fuchs et al., 2016), it is possible that the effects of RAN and number sense on the covariation of reading and arithmetic fluency are mediated. Second, to our knowledge, this is the first study to examine the role of number sense in the covariation of reading and arithmetic fluency. All previous studies had examined the role of counting (Koponen et al., 2007, 2016, 2020; Korpipää et al., 2017).

## Method

### Participants

Our sample consisted of 126 English-speaking children (67 females, 59 males; *M*_age_ = 6.41 years, *SD* = 0.45) followed from the beginning of Grade 1 (October/November, Time 1) to the end of Grade 1 (May/June, Time 2). They were recruited on a voluntary basis (155 children attending Grade 1 in the participating schools were initially invited to participate in the study) from six public elementary schools in Edmonton, Canada. The schools were located in different parts of the city in order to increase the representation of different demographics in our study. Ninety percent of the children were White, 4% East Asian, and 4% Middle Eastern, and 2% Indigenous. None of the children were experiencing any intellectual, emotional, or sensory difficulties (based on school records). Parental and school consent was obtained prior to testing. Ethics approval was also obtained from the University of Alberta (Pro00065133).

### Materials

#### Non-verbal IQ

Non-verbal Matrices from the Cognitive Assessment System-2 (CAS-2; Naglieri et al., 2014) was administered to assess non-verbal IQ. Children were presented with a page containing a pattern of shapes/geometric designs that was missing a piece and were asked to choose among five or six alternatives the piece that would accurately complete the pattern. There were 44 items arranged in terms of increasing difficulty and the test was discontinued after four consecutive errors. A participant's score was the total number correct. Cronbach's alpha reliability in our sample was 0.94.

#### Working Memory

The Backward Digit Span task from Wechsler Intelligence Scale for Children-III (Wechsler, 2002) was used to assess working memory. Children were asked to repeat a sequence of digits in the reverse order. The strings started with only two digits and one digit was added at each difficulty level (the maximum length was seven digits). The task was discontinued when participants failed both trials of a given length. A participant's score was the total number of correctly recalled trials. Cronbach's alpha reliability in our sample was 0.78.

#### Speed of Processing

To assess speed of processing we administered the Matching Numbers task from the CAS-2 (Naglieri et al., 2014). Children were presented with four pages, each consisting of eight rows of numbers with six numbers in each row. The numbers ranged in length from one to six digits. Children were asked to find and underline the two numbers in each row that were the same within a time limit (e.g., 18 22 25 17 33 22). Naglieri et al. (2014) reported test-retest reliability to be 0.75.

#### Phonological Awareness

To assess phonological awareness, we administered the Elision task from the Comprehensive Test of Phonological Processing-2 (Wagner et al., 2013). Children were asked to first listen to a word and then say the word without saying one of its sounds (e.g., Say the word *bold* without saying the/b/sound). The task was discontinued after three consecutive errors and a participant's score was the total number correct (max = 33). Cronbach's alpha reliability in our sample was 0.92.

#### Rapid Automatized Naming

To assess RAN we administered Digit Naming from the RAN/RAS test battery (Wolf and Denckla, 2005). Children were asked to name as fast as possible five digits (2, 4, 5, 7, 9) that were repeated 10 times each and arranged semi-randomly in five rows of ten. Prior to beginning the timed naming, the children were asked to name the digits in a practice trial to ensure familiarity. The time to name all digits was the participant's score. The score was multiplied by −1 to ease the interpretation of our results (a higher score means better performance). Only a few naming errors occurred (mean number of errors was <1) and for this reason they were not considered further. Wolf and Denckla (2005) reported test-retest reliability for Digit Naming to be 0.92.

#### Number Sense

To assess number sense, we administered the Number Sets task (Geary et al., 2009). Children were presented with four pages and each page included a target number at the top (e.g., 5) and sets indicated by two or three linked boxes with Arabic numerals (e.g., 2) and concrete objects (e.g., ▴▴▴). Children were asked to circle all the sets that can be put together to match the target number. The target number of the first two pages was 5 and the time limit was 60 secs per page. The target number of the last two pages was 9 and the time limit was 90 s per page. Signal detection method was used to calculate each child's sensitivity (*d*') in detecting the correct sets based on the number of hits and the number of false alarms (see Geary et al., 2009, for details). Cronbach's alpha reliability in our sample was 0.90.

#### Reading Accuracy

The Word Identification task (Form H) from the Woodcock Reading Mastery Tests—Revised (Woodcock, 1998) was used to assess reading accuracy. Children were asked to read out loud a list of words of increasing difficulty. The task was discontinued after six consecutive errors and a participant's score was the total number correct (max = 104). Cronbach's alpha reliability in our sample was 0.94.

#### Reading Fluency

To assess reading fluency, we administered two tasks: Sight Word Efficiency (SWE; Form A) from the Test of Word Reading Efficiency (Torgesen et al., 2011) and CBM-Maze (Deno, 1985). In SWE, children were presented with a list of 108 words, divided into four columns of 27 words each, and asked to read them as fast as possible. An 8-word practice list was presented first to ensure familiarity with the task demands. The number of words read correctly within a 45 s time limit was the participant's score. Torgesen et al. (2011) reported test–retest reliability of 0.93 for ages 6 to 7. In CBM-Maze, children were exposed to a 96-word passage in which every seventh word was replaced by three options (with the exception of the first sentence that remained intact). The passage was deemed by a group of Grade 1 teachers to be appropriate for this grade level. Children were asked to circle the option that was correctly completing the meaning of each sentence. A participant's score was the number of correct answers minus the number of incorrect answers within a 3 min time limit. Cronbach's alpha reliability in our sample was 0.90. CBM-Maze correlated 0.80 with SWE in our sample. A composite score for reading fluency was subsequently created by averaging the *z*-scores of SWE and CBM-Maze and used in the analyses.

#### Arithmetic Accuracy

The Mathematics Reasoning task from WIAT-III (Wechsler, 2009) was used to assess arithmetic accuracy. The Mathematics Reasoning task is a verbal problem-solving task that measures children's ability to count, identify geometric shapes, and solve single- and multistep word problems. The task was discontinued after four consecutive errors and a participant's score was the total number correct (max = 67). Cronbach's alpha reliability in our sample was 0.88.

#### Arithmetic Fluency

To assess arithmetic fluency, we administered three tasks: Addition Fluency and Subtraction Fluency from WIAT-III (Wechsler, 2009) and Missing Number (Clarke and Shinn, 2004). In Addition and Subtraction Fluency, children were asked to solve as many additions or subtractions as possible within a 1-min time limit by writing their response in the space provided beside each problem. Each subtest included two pages (24 items on each page for a total of 48 problems). A participant's score was the total correct number of additions and subtractions completed within the time limit. Wechsler (2009) has reported test-retest reliability for Addition and Subtraction fluency to be 0.76 and 0.90, respectively. The Missing Number task consisted of three pages of 21 boxes each, arranged in seven rows of three. Each box contained a sequence of four numbers (three numbers and a blank; e.g., 2 3 _ 5). Children were asked to say out loud what number goes in the blank in each box. A participant's score was the total number correct within a 1-min time limit (max = 63). Clarke and Shinn (2004) reported test-retest reliability to be 0.79. Missing Number correlated 0.58 with Addition Fluency and 0.57 with Subtraction Fluency. A composite score for arithmetic fluency was subsequently created by averaging the *z*-scores of these three tasks and used in the analyses.

### Procedure

At Time 1, children were assessed on measures of non-verbal IQ, speed of processing, working memory, phonological awareness, RAN, and number sense. At Time 2, they were assessed on measures of reading/arithmetic accuracy and fluency. At both times, testing was conducted in a quiet room at children's school during school hours by graduate students who received extensive training on test administration and scoring. At both times, testing was completed in one sitting and the order of task administration was fixed across participants. At Time 1, testing lasted ~40 min and at Time 2~30 min.

### Statistical Analyses

**Figure 1:**
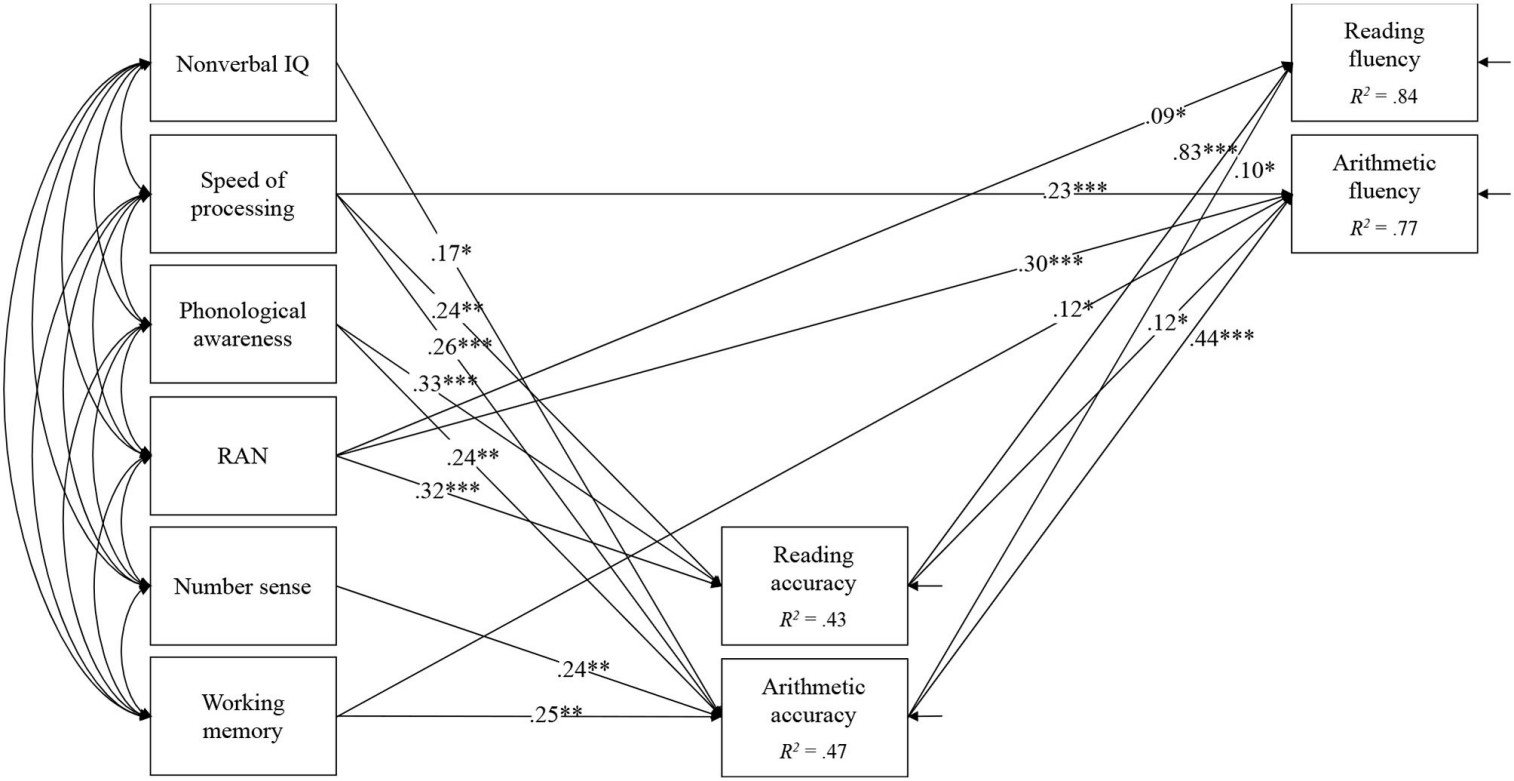
Path model for reading fluency and arithmetic fluency. Non-significant paths are not shown for clarity purposes. *p < 0.05, **p < 0.01, ***p < 0.001.

**Figure 2:**
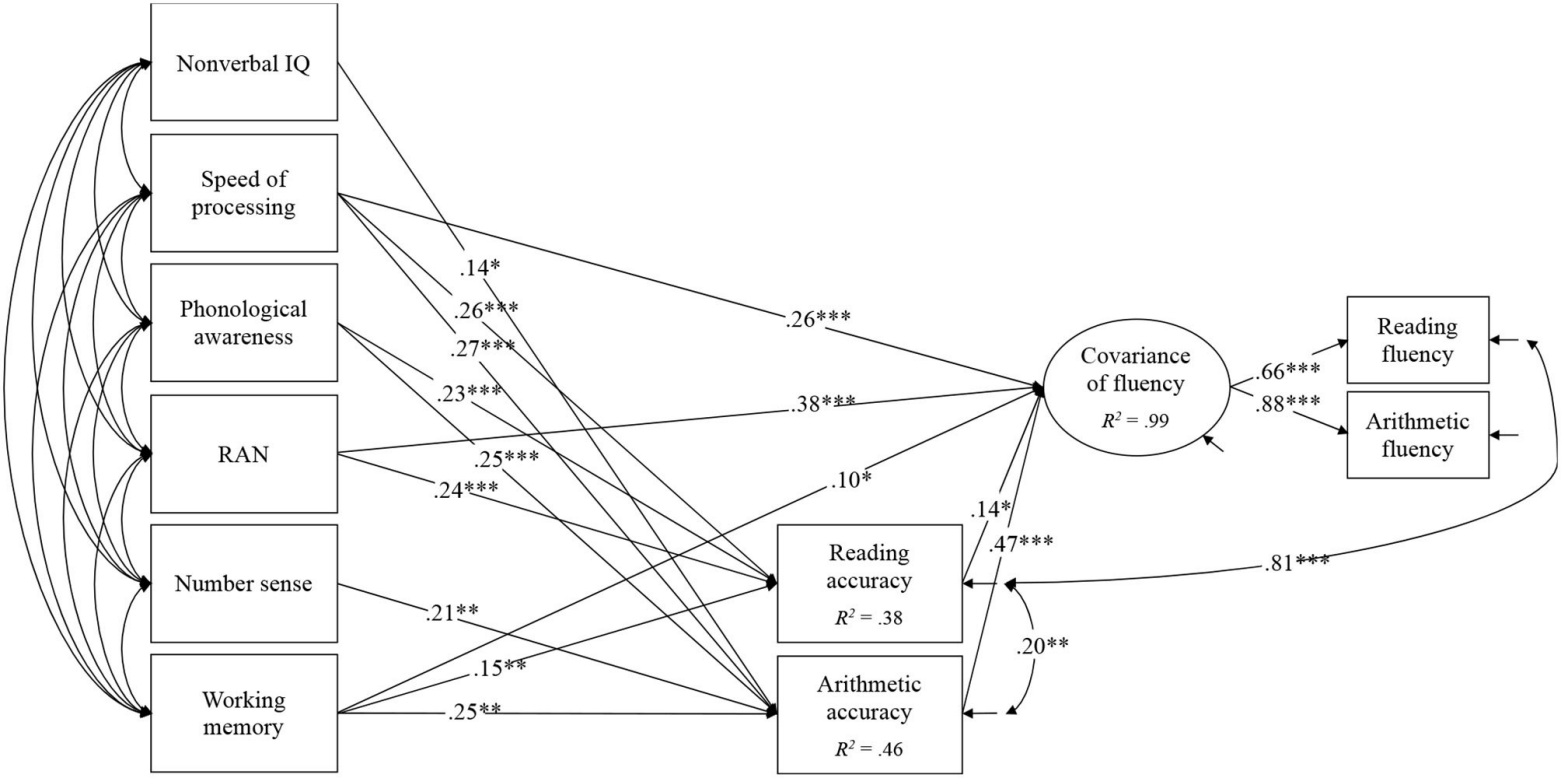
Latent variable model for the covariation of reading fluency and arithmetic fluency. Non-significant paths are not shown for clarity purposes. *p < 0.05, **p < 0.01, ***p < 0.001.

To examine the unique contributions of the cognitive skills to reading fluency, arithmetic fluency, and the covariation of the two, we constructed the following two sets of models. First, a path model for the predictors of reading fluency and arithmetic fluency was constructed (Figure 1). Reading accuracy and arithmetic accuracy were included in the model to test their mediating roles in the relations between the cognitive skills and reading/arithmetic fluency. Non-significant paths were eliminated one at a time from the initial model to evaluate a more parsimonious model with fewer paths. Second, a latent variable model for the covariation of reading fluency and arithmetic fluency was constructed (Figure 2). Additionally, to examine the indirect effect of the cognitive skills on reading fluency, arithmetic fluency, and the covariance factor of fluency through reading accuracy and arithmetic accuracy, we performed a series of mediation analyses using these models. A bias-corrected bootstrapping technique (Hayes and Scharkow, 2013) with 5,000 resamples was used to establish confidence intervals for the indirect effects (Preacher and Hayes, 2008).

All analyses were conducted using Mplus 8.6 (Muthén and Muthén, 1998–2017). Little's Missing Completely at Random test (Little, 1988) showed that our missing data (either due to attrition or to children's decision to discontinue a task) were missing completely at random (χ^2^ = 16.32, *df* = 18, *p* = 0.57), and thus were handled by the full information maximum likelihood estimation. The model fit of each model was assessed using the chi-square value and a set of fit indices: the comparative fit index (CFI), the Tucker-Lewis index (TLI), the root-mean-square error of approximation (RMSEA), and the standardized root-mean-square residual (SRMR). A non-significant chi-square value, CFI and TLI values above 0.95, an RMSEA value below 0.06, and an SRMR value below 0.08 indicate a good model fit (Kline, 2015).

## Results

### Descriptive Statistics and Correlations

The descriptive statistics for the measures used in the study are presented in Table 1. Before conducting any further analyses, we examined the distributional properties of the measures. RAN at Time 1 was positively skewed and log transformation was performed to normalize its distribution. The transformed scores were used in subsequent analyses. In addition, outliers on some measures (defined as more than 3 *SD* above/below the mean) were winsorized to the next non-outlier's score ±1 to avoid overemphasizing their effects on the results (Tabachnick and Fidell, 2012).

**Table 1:** Descriptive statistics for the measures used in the study.

**Measure**	***M***	***SD***	**Range**
Time 1 (beginning of Grade 1)			
Non-verbal IQ (max = 44)	10.61	3.68	0–25
Working memory (max = 12)	3.15	1.24	0–7
Speed of processing (max = 32)	10.28	3.12	4–21
Phonological awareness (max = 33)	10.46	4.42	2–20
RAN	40.90	14.19	21–122
Number sense	2.64	0.67	1.06–3.74
Time 2 (end of Grade 1)			
Word identification (max = 104)	43.96	13.39	5–82
Sight word efficiency (max = 108)	43.77	16.19	0–73
CBM-Maze (max = 13)	4.86	3.46	0–12
Math reasoning (max = 67)	20.59	5.29	11–36
Addition fluency (max = 48)	6.89	5.12	0–26
Subtraction fluency (max = 48)	6.67	4.39	0–25
Missing number (max = 63)	14.95	5.83	3–30

*RAN, rapid automatized naming. With the exception of Number Sense, the descriptive statistics are on the raw scores of each task*.

The zero-order correlations among all of the variables are presented in Table 2. The correlations with the linguistic/number skills (i.e., phonological awareness, RAN, and number sense) ranged from 0.30 to 0.53 for reading fluency and from 0.32 to 0.62 for arithmetic fluency. RAN showed the strongest association with both reading and arithmetic fluency. Additionally, in all instances, the cognitive measures correlated more strongly with arithmetic fluency than reading fluency.

**Table 2:** Correlations between the variables.

	**1.**	**2.**	**3.**	**4.**	**5.**	**6.**	**7.**	**8.**	**9.**
1. T1 NVIQ									
2. T1 WM	0.11								
3. T1 SoP	0.28^**^	0.31^**^							
4. T1 PA	0.17	0.38^**^	0.24^*^						
5. T1 RAN	0.00	0.27^**^	0.35^**^	0.35^**^					
6. T1 NS	−0.02	−0.06	0.23^*^	0.13	0.27^**^				
7. T2 RA	0.19^*^	0.31^**^	0.43^**^	0.50^**^	0.52^**^	0.25^**^			
8. T2 RF	0.22^*^	0.32^**^	0.49^**^	0.39^**^	0.53^**^	0.30^**^	0.91^**^		
9. T2 AA	0.31^**^	0.43^**^	0.50^**^	0.46^**^	0.35^**^	0.32^**^	0.49^**^	0.58^**^	
10. T2 AF	0.17	0.49^**^	0.63^**^	0.48^**^	0.62^**^	0.32^**^	0.62^**^	0.66^**^	0.75^**^

*T1, Time 1; T2, Time 2; NVIQ, Non-verbal IQ; WM, working memory; SoP, speed of processing; PA, phonological awareness; NS, number sense; RA, reading accuracy; RF, reading fluency; AA, arithmetic accuracy; AF, arithmetic fluency*.

* *p < 0.05;*

** *p < 0.01*.

### Structural Models and Mediational Analyses

The path model for reading fluency and arithmetic fluency is shown in Figure 1. The model showed an excellent fit, χ^2^(6) = 4.62, *p* = 0.59, CFI = 1.00, TLI = 1.00, RMSEA = 0, 90%CI [0, 0.10], SRMR = 0.03. The results showed that RAN predicted both reading fluency (β = 0.09) and arithmetic fluency (β = 0.30) even when the effects of reading accuracy and arithmetic accuracy were controlled. Additionally, speed of processing (β = 0.23), and working memory (β = 0.12) had a direct effect on arithmetic fluency.

The model for the cognitive predictors of the covariation of reading fluency and arithmetic fluency is shown in Figure 2. In order to have a well-fitting model, we had to allow the residuals of reading accuracy and reading fluency to covary. The model fit the data very well (χ^2^ = 15.51, *df* = 12, *p* = 0.24, CFI = 0.99, TLI = 0.98, RMSEA = 0.05, 90%CI [0, 0.11], SRMR = 0.04), and the results showed that RAN (β = 0.38), speed of processing (β = 0.26), and working memory (β = 0.10) predicted the covariance factor of fluency over and above the significant effects of reading and arithmetic accuracy. Importantly, the predictor variables accounted for a large amount of variance in the covariance factor (99%).

Finally, the results of the mediation analyses are shown in Table 3. The results showed that speed of processing and phonological awareness had indirect effects on reading fluency, arithmetic fluency, and the covariance of fluency via both reading and arithmetic accuracy. RAN also had indirect effects on the same outcome variables via reading accuracy, while those of non-verbal IQ and number sense were mediated by arithmetic accuracy. Moreover, working memory had indirect effects on reading and arithmetic fluency *via* arithmetic accuracy, and it also had indirect effects on the covariance of fluency via reading and arithmetic accuracy. To summarize, these results indicate that speed of processing and RAN predict reading and arithmetic fluency and the covariation of the two both directly and indirectly through reading and arithmetic accuracy (except the direct effect of speed of processing on reading fluency). Additionally, working memory had direct effects on arithmetic fluency and the covariance factor of fluency over and above its indirect effects via reading and arithmetic accuracy. In contrast, phonological awareness and number sense predict reading fluency, arithmetic fluency, and the covariance factor of fluency only indirectly through the accuracy measures.

**Table 3:** Indirect effects of the cognitive predictors on reading fluency, arithmetic fluency, and the covariance of fluency.

**Path**	**Estimate**	**Bootstrapped 95% CI**
Reading and arithmetic fluency		
NVIQ → AA → RF	0.017	[0.002, 0.050]
NVIQ → AA → AF	0.073	[0.017, 0.143]
SoP → RA → RF	0.195	[0.081, 0.312]
SoP → RA → AF	0.029	[0.006, 0.067]
SoP → AA → RF	0.026	[0.003, 0.069]
SoP → AA → AF	0.113	[0.055, 0.187]
PA → RA → RF	0.269	[0.148, 0.378]
PA → RA → AF	0.041	[0.006, 0.092]
PA → AA → RF	0.024	[0.006, 0.057]
PA → AA → AF	0.106	[0.037, 0.188]
RAN → RA → RF	0.266	[0.150, 0.376]
RAN → RA → AF	0.040	[0.009, 0.087]
NS → AA → RF	0.024	[0.002, 0.063]
NS → AA → AF	0.103	[0.042, 0.177]
WM → AA → RF	0.025	[0.005, 0.062]
WM → AA → AF	0.109	[0.048, 0.189]
Covariance of fluency		
NVIQ → AA → CoF	0.066	[0.003, 0.136]
SoP → RA → CoF	0.037	[0.008, 0.081]
SoP → AA → CoF	0.129	[0.063, 0.209]
PA → RA → CoF	0.032	[0.005, 0.083]
PA → AA → CoF	0.118	[0.040, 0.207]
RAN → RA → CoF	0.035	[0.009, 0.080]
NS → AA → CoF	0.098	[0.039, 0.167]
WM → RA → CoF	0.021	[0.004, 0.052]
WM → AA → CoF	0.115	[0.052, 0.196]

*NVIQ, Non-verbal IQ; SoP, speed of processing; PA, phonological awareness; NS, number sense; WM, working memory; RA, reading accuracy; AA, arithmetic accuracy; RF, reading fluency; AF, arithmetic fluency; CoF, covariation of fluency*.

## Discussion

The purpose of this study was to examine the shared and unique predictors of reading and arithmetic fluency and whether their effects are mediated by reading and mathematics accuracy. To this end, we used two slightly different approaches: First, we used reading and arithmetic fluency as separate outcomes in the same model. Our findings showed that only RAN Digits predicts both outcomes over and above the effects of reading and mathematics accuracy. Speed of processing and working memory predicted only arithmetic fluency. In regard to RAN Digits, our finding replicates those of previous studies (Koponen et al., 2007, 2013, 2016; Hornung et al., 2017) and suggests that word reading fluency and arithmetic fact retrieval rely on how quickly one could access the phonological representations of words or numbers. Notably, this is independent of the effects of speed of processing. The unique effect of RAN Digits on reading and arithmetic fluency over and above the effects of speed of processing has already been documented (e.g., Georgiou et al., 2009; however, see also Georgiou et al., 2013; Cui et al., 2017). The fact that processing speed and working memory predicted only arithmetic fluency may be due to the strong effects of reading accuracy on reading fluency that left very little room for other variables to make any significant contributions. In fact, when we reran our analyses without reading accuracy, both speed of processing and working memory predicted reading fluency. However, this finding may also reflect the fact that both speed of processing and working memory tasks involved processing of numbers and this brought them closer to arithmetic fluency.

Second, we tested a model in which the cognitive-linguistic skills were used as predictors of the covariation of reading and arithmetic fluency. This approach allows us to examine what skills predict what is shared between reading and arithmetic fluency. For example, if these two are related because they both require speeded responses, then speed of processing should predict their covariation. Our findings were slightly different than those of the first approach. More specifically, RAN, speed of processing, and working memory were unique predictors of the covariation of reading and arithmetic fluency. This suggests first that the cognitive base of reading and arithmetic fluency consists of multiple cognitive processes. Obviously, both outcomes require speeded responses (hence the effects of speed of processing). However, on top of that, they also require quick access and retrieval of phonological representations stored in long-term memory (hence the effects of RAN and working memory). Second, it shows that some cognitive processes (i.e., speed of processing and working memory) might be related more to what reading and arithmetic share than what is unique to them (see Figure 1), when it is used as a separate outcome in the analyses. This implies that depending on the approach used researchers may draw slightly different conclusions.

Phonological awareness and number sense contributed to the covariance of reading and arithmetic fluency indirectly through the effects of reading and mathematics accuracy. The strong connection between phonological awareness and reading accuracy is not surprising and has been reported in several previous studies (see e.g., Melby-Lervåg et al., 2012; Ruan et al., 2018, for evidence from meta-analyses). Successful decoding relies on children's ability to blend the sounds in words. However, perhaps less expected is the significant effect of phonological awareness on mathematics accuracy. Previous studies on the relation between phonological awareness and mathematics skills provided mixed findings (for significant effects see Cirino et al., 2018; Zhang and Lin, 2018; Yang and McBride, 2020 for non-significant effects see Durand et al., 2005; Koponen et al., 2016; Peterson et al., 2017; Yang et al., 2021). An explanation for the mixed findings may relate to the type of mathematics task used as an outcome in different studies. According to the triple-code model (Dehaene, 1992; Dehaene et al., 2003), three types of codes are used in numerical processing: a visual code, a verbal code, and an analog magnitude code. Phonological awareness may be predictive of mathematics tasks like Mathematic Reasoning that include more items requiring processing of verbal codes than some other tasks. This explanation is independent of the complexity of the mathematics problems and whether the solution to a given problem can be retrieved directly from memory. For example, De Smedt et al. (2010) showed that phonological awareness was a significant predictor of only arithmetic problems with a small problem size and concluded that this is likely due to the fact that these problems can be solved by rapid retrieval of the problem's solution from long-term memory. This explanation is problematic as it has also been used to explain why RAN predicts more strongly arithmetic fluency tasks such as addition and multiplication fluency (but not subtraction or division fluency) that involve rapid retrieval of an answer from memory (e.g., Georgiou et al., 2013, 2020; Cui et al., 2017). In our study, phonological awareness predicted arithmetic accuracy but not fluency. This suggests that it is not the rapid access but the integrity/quality of the accessed phonological codes that matters in this case.

In contrast to phonological awareness, number sense appears to have a more domain specific contribution as it predicted only mathematics accuracy (see Jordan et al., 2010; Slot et al., 2016, for a similar finding) and through the effects of mathematics accuracy the covariance of reading and arithmetic fluency. This suggests that in the early phases of reading and mathematics development, there is a set of skills such as non-verbal IQ and number sense that may exert domain specific rather than domain general effects.

Some limitations of the present study should be reported. First, we used single measures of each predictor variable. Obviously, administering more tasks would strengthen each construct, but given the time restrictions associated with assessing young children, we had to make a tough choice between covering more constructs with a single task and assessing fewer constructs with more measures. We opted for the former. Second, our study included Grade 1 children and our findings may not generalize to other grade levels. This is important to note because the effects of some cognitive-linguistic skills (e.g., RAN) on reading and mathematics may vary across grade levels (e.g., Araújo et al., 2015). Third, we did not include counting in our study. A pilot study we ran prior to collecting these data produced ceiling effects in counting and for this reason we did not assess it. This does not allow us to compare our findings to those of previous studies that assessed counting (Koponen et al., 2007, 2016; Korpipää et al., 2017). Fourth, we did not administer a measure of vocabulary. Finally, our RAN, speed of processing, and working memory tasks involved numbers and this may have inflated their relation with mathematics accuracy and fluency. A future study should replicate our findings using also neutral RAN, speed of processing, and working memory tasks.

## Conclusion

Do reading and arithmetic fluency share a similar cognitive base? Our findings add to those of previous studies (e.g., Koponen et al., 2007, 2020; Korpipää et al., 2017; Cirino et al., 2018; Balhinez and Shaul, 2019) and show that the answer is not straightforward. On the one hand, there was a set of cognitive skills (i.e., RAN, speed of processing, and working memory) that exerted both a direct and an indirect effect on the covariance of reading and arithmetic fluency. For these processing skills we can say with some confidence that they are part of a cognitive base that supports both reading and arithmetic fluency. On the other hand, there was a second set of processing skills (i.e., non-verbal IQ, phonological awareness, and number sense) that predicted the covariation of reading and arithmetic fluency through the effects of reading and mathematics accuracy. In fact, number sense and non-verbal IQ predicted only mathematics accuracy, which suggests that some processing skills might be uniquely associated with mathematics (see Slot et al., 2016; de Megalhães et al., 2021; for a similar finding). Taken together, these findings suggest that reading and arithmetic fluency do not rely on a single cognitive process, but rather on a broader network of linguistic and general cognitive abilities. Future studies should replicate our findings following the same children over a longer period of time and in different languages.

## Data Availability Statement

The raw data supporting the conclusions of this article will be made available by the authors, without undue reservation.

## Ethics Statement

The studies involving human participants were reviewed and approved by Ethics Board of the University of Alberta. Written informed consent to participate in this study was provided by the participants' legal guardian/next of kin.

## Author Contributions

GG and RP designed the study. GG prepared the data for the analysis and wrote the introduction, method, and discussion sections of the manuscript. TI ran the analyses and wrote the results section of the manuscript. All authors interpreted the data and discussed the results.

## Conflict of Interest

The authors declare that the research was conducted in the absence of any commercial or financial relationships that could be construed as a potential conflict of interest.

## Publisher's Note

All claims expressed in this article are solely those of the authors and do not necessarily represent those of their affiliated organizations, or those of the publisher, the editors and the reviewers. Any product that may be evaluated in this article, or claim that may be made by its manufacturer, is not guaranteed or endorsed by the publisher.

## Footnotes

^1^Because they assessed reading and arithmetic fluency in both Grades 1 and 7, they created both a time-specific covariance factor and a time-invariant covariance factor of reading and arithmetic fluency across the two grades.
